# Efficient Micropropagation of Highly Economic, Medicinal and Ornamental Plant *Lallemantia iberica* (Bieb.) Fisch. and C. A. Mey

**DOI:** 10.1155/2014/476346

**Published:** 2014-08-27

**Authors:** Fethi Ahmet Ozdemir, Mehmet Ugur Yildirim, Mahsa Pourali Kahriz

**Affiliations:** ^1^Department of Molecular Biology and Genetics, Faculty of Sciences, Bartin University, 74100 Bartin, Turkey; ^2^Department of Field Crops, Faculty of Agriculture, Ankara University, Diskapı, 06110 Ankara, Turkey

## Abstract

*Lallemantia iberica* (Bieb.) Fisch. and C. A. Mey is high valued annual ornamental and medicinal plant from Lamiaceae family that prefers dry sunny hillsides, roadsides, slopes, and fallow fields over an altitude of 500–2150 m. It bears beautiful white flowers and bloom from April to June each year. This study reports *L. iberica* micropropagation using cotyledon node explants isolated from 15-day-old *in vitro* regenerated plantlets. The cotyledon node explants were cultured on MS medium containing 0.50, 1.00 plus 2.00 mg/L BAP, 0.00, 0.01, and 0.02 mg/L NAA. Maximum shoot regeneration was noted on MS medium containing 0.50 mg/L BAP. Well-developed micropropagated shoots were rooted on MS medium containing 1.00 mg/L IBA. The rooted plants were easily hardened in the growth chamber and acclimatised in greenhouse.

## 1. Introduction

*Lallemantia iberica*, family Lamiaceae, is a beautiful ornamental annual plant that grows over large area in Turkey. The family contains 236 genera and about 7173 species that grow on wide area extending from the Asia minor to the Caucasian countries, the Middle East, and the Central and the Southern Europe over an altitude of 500–2150 m and blooms during April to June each year [1–3].

Clonal propagation of medicinal plants requires development of successful* in vitro* plant tissue culture techniques. A review of literature shows development of number of protocols for large number of aromatic and medicinal plants [4–7].

It is economically important and has high ornamental value, as it is used in arid landscaping and urban horticulture at various places in Turkey. It is also grown for use in food, lighting, pharmaceutical purposes [5], and traditional medicinal systems [8] as stimulant, diuretic, and expectorant [9, 10]. Sometimes, it is also used to treat some nervous, hepatic, and renal diseases [11]. The seed contains up to 30% o f a drying oil [12]. Nori-Shargh et al. [13] observed that its oil has 33.7% germacrene, 19.0% 3-carene, 12.8% isocaryophyllene, 11.1% sabinene, 6.5% terpinene acetate, and 4.4% limonene.

Cotyledon node has been used by many researchers for direct shoot regeneration as it is faster regeneration approach to obtain whole plant [1, 14–17]. There is need to propagate this plant for wider uses through traditional and biotechnological approaches. There has been no report on* in vitro* propagation of* L. iberica*. Therefore, this study aimed to establish a viable axillary shoot regeneration protocol using cotyledon node as explant.

## 2. Materials and Methods

The seeds and plant samples of* L. iberica *were obtained from the surroundings of Elazig subdivision in the Elazig province of Turkey and were identified in the section plant taxonomy of the Department of Biology, Firat University, Elazig, Turkey, where voucher sample of this plant was also deposited.

The seeds were surface sterilized using 100% bleach (5% NaOCl Ace, Turkey) followed by rinsing 3 × 5 min with sterile distilled water. Thereafter, the seeds were cultured on agar solidified MS medium [18] containing 3% sucrose for germination. The seeds began to germinate after one week. The cotyledon node explants were obtained from the seedlings under aseptic conditions and cultured on MS medium containing 0.50, 1.00, and 2.00 mg/L BAP (6-benzylaminopurine) and 0.00, 0.01, and 0.02 mg/L NAA (*α*-naphthalene acetic acid) [9 combinations] supplemented with 3% (w/v) sucrose and 0.6%  (w/v) plant agar (Duchefa) for shoot regeneration and multiplication.

Well-developed shoots induced on cotyledon node explant were used in the rooting experiment. These shoots were rooted on MS medium containing 1.00 mg/L IBA.

All regeneration and rooting media were autoclaved for 20 min at 1.4 kg cm^−2^. The pH of all media was adjusted to 5.7 ± 0.1 with 1 N NaOH or 1 N HCl. They were poured into 200 mm 10 × 10 mm culture boxes. All cultures were grown at 24 ± 2°C with a 16 h light photoperiod. Light was supplied at intensity of 45 *μ*mol m^−2^ s^−1^ by Phillips day light lamps.

After acclimatization of well-rooted and healthy plantlets, agar was removed from their roots under running tap water followed by transfer to pots containing peat moss, which were covered with transparent polyethylene bags to create a high relative humidity during initial stages of prehardening and kept at 24° ± 1°C + 80% relative humidity in growth chamber. Each of the developing explants was transferred to 5.00 litre pots containing 4.50 litres of peat moss for 15–18 d. Thereafter, the transparent polyethylene bags were gradually opened and removed in 10–13 d after achieving hardening. These plantlets were maintained in greenhouse for growth, development, flowering, and morphological observations during posthardening or acclimatisation.

Each replication contained 10 explants and each treatment was replicated thrice.

Experimental data given in percentages were subjected to square root (√*X*) transformation before statistical analysis following Snedecor and Cochran [19]. The experimental data were subjected to one-way analysis of variance and the post hoc Tukey's *b* test using IBM SPSS for Windows v. 20.

## 3. Results

The data pertaining to regeneration of* L. iberica *was subjected to variance analysis. The result showed that the combinations and rates of plant growth regulators used in the study sharply affected callus induction percentage (*F* = 4.62, df = 8; *P* < 0.01). No callus induction was noted on MS medium containing 0.50 mg/L BAP (Table 1). Callus induction on the rest of the treatments ranged from 13.33 to 66.67%. Maximum callusing was induced on MS medium containing 0.50 mg/L BAP + 0.02 mg/L NAA followed very closely by 1 mg/L BAP. The rest of the explants induced maximum callusing of 33.33% was noted on rest of the treatments.

The result showed that the combinations and rates of plant growth regulators used in the study sharply affected shoot induction percentage (*F* = 3.80, df = 8; *P* < 0.01). 100.00% shoot regeneration was noted on MS medium containing 0.5 mg/L BAP. The rest of the plant growth regulator combinations affected shoot regeneration in range of 33.33–86.67%. No shoot regeneration was noted on MS medium (control).

**Figure 1:**
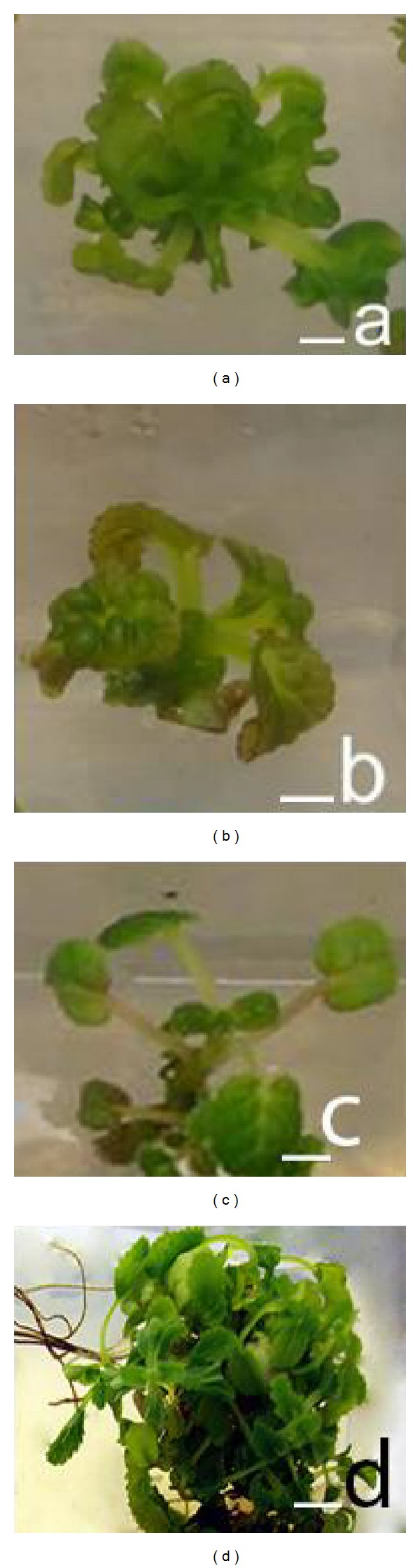
Mass propagation of* L. iberica*: (a, b) hyperhydricity on developing shoots cultured on MS medium containing 0.5 mg/L BAP, (c) recovery of hyperhydric shoots on MS medium, and (d) developing and growing rooted shoots with profusely leaved branches on 1 mg/L IBA (Bar, (a, b) = 1 cm, (c) = 0.90 cm, and (d) = 0.4 cm).

The result further showed that combinations and rates of plant growth regulators used in the study also affected mean number of shoots per explant (*F* = 14.55, df = 8; *P* < 0.01). Number of shoots per explant ranged from 3.33 to 17.80. Maximum shoot regeneration was noted on 0.5 mg/L BAP. However, these shoots were hyperhydric at the initial stages of growth (Figures 1(a) and 1(b)). It was followed by significantly reduced number of 10.00 and 9.47 shoots per explant on MS medium containing 2.00 mg/L BAP and 0.50 mg/L BAP + 0.01 mg/L NAA, respectively. The rest of the cultures regenerated on variants of BAP with or without 0.01 or 0.02 mg/L NAA regenerated significantly reduced number of shoots per explant. These shoots never increased beyond 4.87 in upper bound and decreased below 3.33 shoots per explant in lower bound. No shoots were noted on MS medium. All hyperhydric shoots were cut to single shoots and transferred to MS medium to recover hyperhydricity. The shoots regenerated new leaves that were not hyperhydric; however, old leaves died. These shoots with developing new leaves were not difficult to grow on MS medium, where they grew sturdy healthy growing shoots (Figure 1(c)).

Well-developed shoots regenerated on MS medium containing 0.5 mg/L BAP were rooted on MS medium containing 1.00 mg/L IBA, where they developed profuse rooting and shoots (Figure 1(d)). No signs of hyperhydricity were noted on any plant that was acclimatised in the greenhouse. Each of the greenhouse grown plants showed visible signs of growth; they flowered and did not show any morphological variation when compared with non-tissue cultured plants.

The results showed that it was possible to multiply this plant using cotyledon node explant on MS medium containing different concentrations of 0.5 mg/L BAP.

## 4. Discussion

As most of the plants are not cultivated or micropropagated under similar conditions, they vary in their characteristics. Secondary metabolites vary from season to season and developmental stage of the plant. Sustainable production of drugs in the pharmaceutical industry depends on continuous supply of healthy material to which plants provide a major contribution [20, 21]. Developing reliable propagation protocols of these economically important medicinal plants could help beneficially.

The study made use of 9 different combinations of plant growth regulators for regeneration from cotyledon nodes of* in vitro *regenerated seedlings. The results indicated that MS medium without growth regulators was not suitable for regeneration. The explant did not swell or induced any callus. The results are in agreement with the previous findings of Xiang et al. [22], Gnamien et al. [23], and Liu and Chen [24], who described callus induction from immature embryos, hypocotyl, and leaf segments of* Swertia mussotii*, another plant of Labiatae family, and failed to obtain any regeneration on MS medium. However, all concentrations of BAP with 0.00, 0.01, and 0.02 mg/L NAA induced shoot regeneration variably in plant growth regulator concentration and combination dependent manner. The results of the study also very clearly indicated that MS medium containing 0.50 mg/L BAP was optimum for shoot bud initiation and shoot regeneration. However, the cotyledon nodes cultured on the rest of treatments induced moderate to profuse callusing that was partially inhibitory in shoot regeneration. The results of this study indicated that shoot regeneration was partially inhibited by callus regeneration. Maximum shoot regeneration percentage was induced on 0.50 mg/L BAP, where there was no callus induction.

Callus induction inhibited shoot regeneration percentage variably. Hundred percent (100.00%) callusing was not noted on any regeneration medium. It varied variably due to interaction among physical-chemical factors affecting tissue cultured explants. The results are in agreement with He et al. [25], who noted different treatments and combinations of BAP + NAA that induced variable number of shoots on calli induced on cotyledon node of* Swertia mussotii* after 6 weeks, once calli were transferred to the regeneration medium. Similarly, Dong et al. [26] used callus to induce adventitious buds on rosemary that grew to intact plants.

This variation was more evident, when the results were related with the number of shoots per explant. Maximum number of shoots per explant was noted on MS medium containing 0.50 mg/L BAP with or without any concentration of NAA, when no callus was induced. Each increase in the BAP concentration was inhibitory and showed corresponding sharp decrease in the number of shoots per explant. MS medium containing 0.50, 1.00, and 2.00 mg/L BAP with 0.01 mg/L NAA had statistically similar effect on number of shoots per explant. Excluding this, the rest of the cultures induced 3.33 to 4.87 shoots per explant. The results of this study are in agreement with Patnaik and Debata [27], Smolenskaya and Ibragimova [28], Sivanesan and Jeong [29], and Yildirim [30], who emphasize that a combination of cytokinin and auxins especially BAP with low level of auxin is necessary for shoot regeneration and morphogenesis. The results of the present study also revealed that shoot bud induction and shoot regeneration from cotyledon node improve with decreasing levels of BAP and are inhibited with increasing levels of BAP in the regeneration medium in significant relation to levels of NAA. Safdari and Kazemitabar [31] do not agree with this conclusion and report unsuitability of BAP at low concentrations, for shoot regeneration from nodal segments of* Portulaca grandiflora*.

*L. iberica *cotyledon node explant reacted variably with BAP-NAA. Callusing was inhibited variably in the presence of NAA. However, concentrations of 1.00 and 2.00 mg/L BAP were equally good for callus induction and shoot regeneration.

The* in vitro *regenerated plantlets could be rooted and acclimatized without any difficulty. The results of this study are in agreement with Khawar et al. [2], Sevimay et al. [1], and Daneshvar-Royandezagh et al. [32], who emphasize the role of auxins in* in vitro *rooting of micropropagated medicinal plants.

This is the first report of plantlet regeneration from cotyledon node explants of* L. iberica. *In conclusion, this study establishes an efficient regeneration protocol for micropropagation of this plant with large potential for use in pharmaceutical industry using young cotyledon node explants. This technique should be a very useful tool for large scale propagation strategies and will serve importantly future* Lallemantia* agronomists and breeders.

## Figures and Tables

**Table 1:** Effects of various concentrations of BAP-NAA on shoot regeneration from cotyledonary node explant of *L. iberica*.

Treatments		Callus induction percentage	Shoot regeneration percentage	Number of shoots per explant
BAP (mg/L)	NAA (mg/L)			
0.50	0.00	0.00^f^	100.00^a^	17.80^a^
0.50	0.01	26.67^c^	73.33^d^	9.47^b^
0.50	0.02	66.67^a^	33.33^e^	3.33^c^
1.00	0.00	60.00^a^	66.67^d^	4.13^c^
1.00	0.01	20.00^d^	80.00^c^	4.87^c^
1.00	0.02	33.33^b^	66.67^d^	4.60^c^
2.00	0.00	13.33^e^	86.67^b^	10.00^b^
2.00	0.01	33.33^b^	66.67^d^	4.87^c^
2.00	0.02	20.00^d^	80.00^c^	4.87^c^
MS medium (control)		0.00^f^	0.00^f^	0.00^d^

Means of values showed in each column followed by different letters are statistically not similar using Tukey's test at 0.01 level of significance.
